# 

**Published:** 1915-06-26

**Authors:** 


Mr. E. G. Easton has been elected a member of the Epileptic Colony and Horton Estate Sub-Committee of the London County Council.

Dr. R. P. Weldon has resigned his appointment as fifth assistant medical officer at Long Grove Mental Hospital, on obtaining an appointment in the Colonial Medical Service.

### Buildings Contemplated and in Progress.

BEXHILL.—The Town Council have decided not to build the whole of the new isolation hospital, as previously determined, but only to carry out the sewerage works, and to provide an observation block at a cost of £1,377.

DEAL.—At the annual meeting of the Victoria Hospital Committee it was announced that an offer of £3,000 had been received towards a proposed enlargement scheme.

HESTON.—The Middlesex County Council have instructed Mr. H. G. Crothall, the county architect, to prepare plans for a new asylum at Heston.

ST. AUSTELL.—The new infirmary at the workhouse, built at a cost of £4,000, has been formally opened. Mr. B. C. Andrew was the architect.

SKIPTON.—Mr. J. Hartley, architect, Skipton, has prepared a scheme for the extension of the hospital. The estimated cost is £1,000.

STONEBRIDGELEES.—The health committee of the Cumberland County Council have under consideration plans and estimates prepared by the county architect for a tuberculosis hospital at Stonebridgelees.

TEDDINGTON.—The Urban District Council have decided, subject to an agreement being arranged, to join the Staines Rural District Council with a view to providing accommodation for smallpox cases.

TOTTENHAM.—The Urban District Council have agreed to purchase a fever hospital site in White Hart Lane, and have referred to the Finance Committee the question of applying to the Local Government Board for sanction to a loan of £8,736 to cover the cost.

WHITLEY.—The Dewsbury Corporation have instructed the surveyor to obtain tenders for alterations and additions at the Whitley Hospital. The Local Government Board have approved the scheme.

YORK.—The Health Committee recommend the Corporation to instruct the City Engineer to prepare detailed plans and estimates of a modified scheme for the extension of the fever hospital.

BARNSLEY.—The Mount Vernon Sanatorium, Barnsley, has been acquired and equipped at a cost of £6,500 and formally opened.

**RATES OF SUBSCRIPTION:** Twelve months, 6/6; Six months, 4/-; Three months, 2/-, post free to any address in the United Kingdom. Abroad, Twelve months, 8/8; Six months, 5/-; Three months, 2/6, post free.—Address The Subscription Dept., "THE HOSPITAL," The Hospital Building, 28/29 Southampton Street, Strand, London, W.C.

